# Traditional ecological knowledge and local language amid a gold rush: age- and gender-related variation in the Awiakay community, Papua New Guinea

**DOI:** 10.1186/s13002-026-00975-5

**Published:** 2026-11-09

**Authors:** Darja Hoenigman, Aung Si

**Affiliations:** 1 School of Culture, History and Language, The Australian National University, Coombs Building #9, Fellows Road, Canberra, ACT 2601 Australia; 2 Institute for Linguistics, University of Cologne, Albertus-Magnus-Platz, 50923 Cologne, Germany

**Keywords:** TEK, Awiakay, Papua New Guinea, Gold rush, Artisanal gold mining, East Sepik Province, Arafundi, Knowledge transmission, Papuan languages

## Abstract

**Background:**

The start of a gold rush on the lands of the Awiakay (Papua New Guinea) in 2020 set in motion a range of adverse changes in social relations, community life, and everyday practices. Accompanied by a shift towards the lingua franca Tok Pisin, these changes may be disrupting the transmission of traditional ecological knowledge (TEK) to the younger generation. Here, we present the results of a study aimed at quantifying the TEK of Awiakay speakers – male and female – across three age groups.

**Methods:**

The ability to name culturally relevant ethnotaxa was used as a proxy for a respondent’s level of TEK. Sixty-one Awiakay speakers were individually presented with colour photographs of 80 ethnotaxa, including plants, birds, bats and other small mammals, and asked to name them in Awiakay. Responses (including those given only in Tok Pisin, or those consisting solely of cultural/ethnobiological information, but no name) were noted, and analysed statistically using ANOVA, t-test and correlation analysis.

**Results:**

Males performed better overall (51.4% correct Awiakay names, vs. 37.9% for females), but this was driven primarily by the former’s superior knowledge of birds and bats. There was also an increase in performance with age: among males, the scores plateaued rapidly, whereas females showed a more gradual increase in scores. Lack of knowledge of individual ethnotaxa could be related to recent lifestyle changes. Among the children, the lowest scorers tended to belong to families that were most intensely involved in the gold rush.

**Conclusions:**

The results suggest that the gold rush is likely disrupting the transmission of TEK in the Awiakay community. Further research is required to conclusively disambiguate the effects of gold panning from the normal acquisition of TEK among young children.

## Introduction

For the vast majority of communities living in non-urban settings, people’s traditional ecological knowledge (TEK) permeates many, if not all, spheres of life, from subsistence [1] to spirituality [2] and from health [3] to leisure [4]. TEK is an essential component of local food security [5, 6], and it also equips a community with the tools necessary for adapting to climate change [7]. Consequently, the erosion of TEK can have far reaching negative impacts on communities, particularly those that are marginalised and/or impoverished.

Reports of TEK loss from around the world are frequently encountered in the ethnobiological literature. Often, one finds that a number of factors are blamed for the breakdown of TEK transmission in any given community, and for the subsequent paucity of TEK among younger generations [8, 9]. These commonly include formal education, language loss, market forces, environmental degradation and migration [10, 11]. As individuals and social groups within a community can be affected by these factors to different degrees, there is often significant variation in TEK within communities [12–14], which can be readily detected by means of various research methodologies [15]. The presence of specific types of TEK variation may provide clues regarding the causes of the variation, and perhaps even the causes of TEK erosion in a particular community. Furthermore, external geographical or environmental factors such as remoteness (distance from government centres/services) and forest cover may also have an impact on the loss or preservation of TEK and language, but only in certain situations [16–18]. Finally, the endangerment or loss of small languages (accompanied by a shift towards a more dominant language) can interact with TEK loss and local biodiversity in complex ways [13, 19–21]. While it is widely accepted that erosion of one or more of the above (language, TEK, biodiversity) is often accompanied by the loss of the others, the precise mechanisms that link these phenomena together require further investigation.

**Fig. 1:**
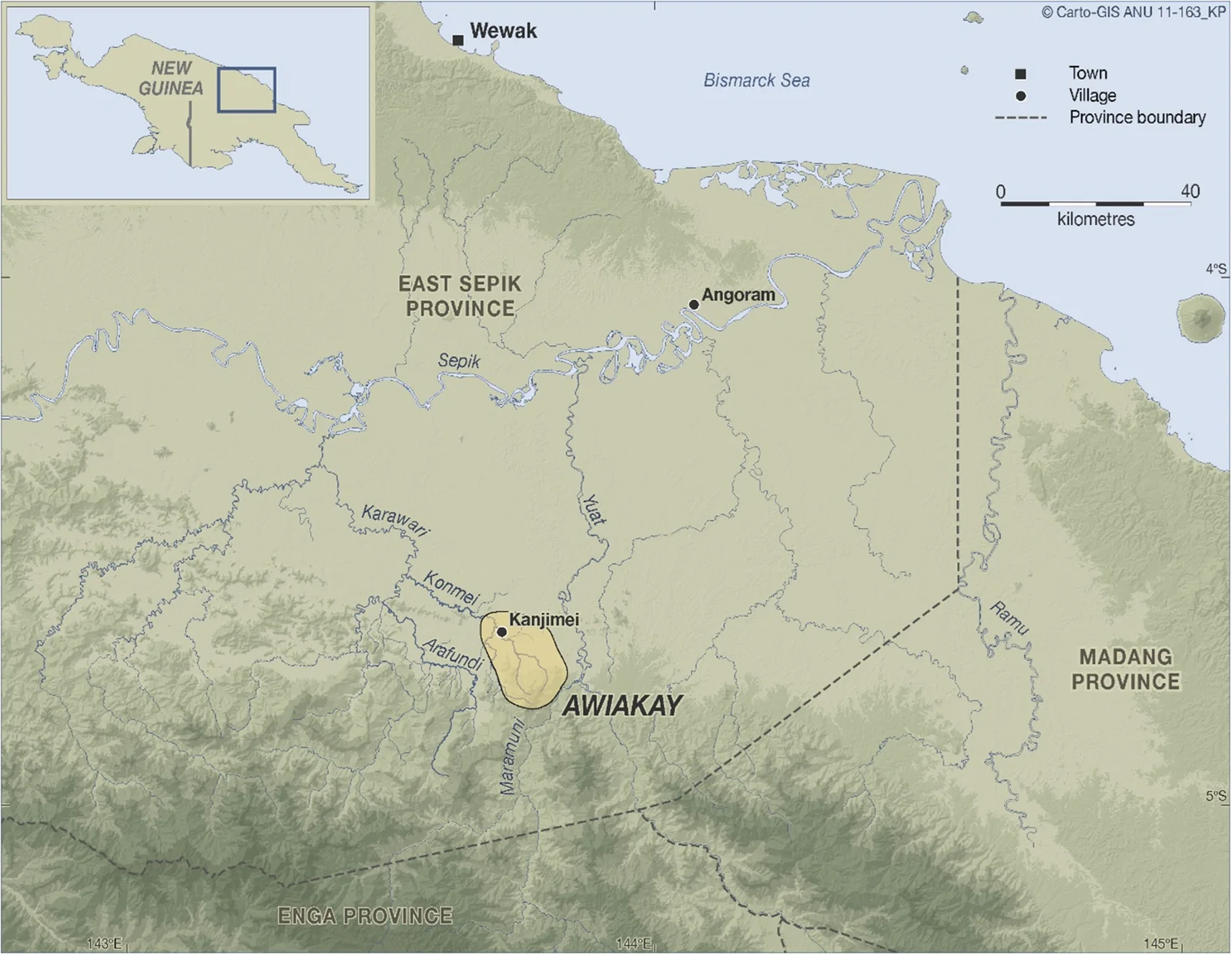
Map of East Sepik Province, Papua New Guinea, showing the location of the study site

With these concerns in mind, the current study investigates variation in TEK – the knowledge itself, and the language in which it is encoded – among the inhabitants of the remote^1^ village of Kanjimei, East Sepik Province, Papua New Guinea (PNG) (Fig. 1). Kanjimei is home to the Awiakay people, a society of about 500 individuals. Their language, also called Awiakay, belongs to the small Arafundi family [22, 23]. All adults and teenagers are bilingual in Awiakay and Tok Pisin (henceforth, TP), the local lingua franca (as well as one of the three national languages of PNG). The current study involved the participation of both men and women, as well as children of different ages. The inclusion of females in population-level ethnobiological studies is particularly important, as men and women can have differing roles, competencies and knowledge bases with respect to interactions with living organisms [24]. Such inclusive research can also help dispel gender-related stereotypes and assumptions that have no empirical basis, as discussed in Brightman [25].

### Linguistic situation

Language is known to play an important role in the maintenance of biocultural diversity [26], and there have been calls to preserve minority and indigenous languages in order to protect the innumerable forms of TEK around the world [21], as the former serves as a repository for the latter. When the first author started working in Kanjimei in 2004, all children were fluent in Awiakay. While most people were bilingual, there were still a few adults in the community who could not speak TP at all. The use of TP in the village was limited to the domains of Church and government-related contexts – both connected with the outside world and authority. TP was also used to communicate with any visitors to the village, including those from neighbouring communities, since none of them speak Awiakay. In everyday contexts, TP was used in swearing, in ordering others around (especially in short instructions to children), and in situations when a speaker wanted to prevail in a communicative act. This manifested in code-switching, in particular in intra-village fights and public oratory [22, 27]. Over the years, the use of TP in the village has increased, mainly due to greater mobility, which resulted in an influx of new things, concepts and ideas from outside, which are connected with TP (Table 1). The use of the Awiakay language did not decline straight away, but was even consciously adapted to new circumstances (see Hoenigman [28] and [22] for a creation of Awiakay ‘hidden talk’ register). In the last couple of years, however, after the onset of a gold rush (see below for more details), TP has become prevalent among small children, and there is a possibility that TEK might also be declining, as is the case in many language communities of PNG.

**Table 1:** Variation in the use of Awiakay and Tok Pisin^1^

	2004	2009–2018	2025
*Knowledge of Awiakay and Tok Pisin*			
Men	All bilingual	All bilingual	All bilingual
Women	Some cannot speak/understand TP	All bilingual	All bilingual
Children	Acquire TP after Awiakay	Acquire TP after Awiakay	Small children monolingual in TP
*TP in Interactions with outsiders*			
Men	Occasionally	Occasionally	Daily in gold camps
Women	Seldom	Occasionally	Daily in gold camps
Children	NA	NA	Daily in gold camps
*Language used in various registers in the village*			
Public speeches	Awiakay + TP	Awiakay + TP	Awiakay + TP
Intra-village disputes	Awiakay + TP	Awiakay + TP	Awiakay + TP
Church	TP + Awiakay	TP + Awiakay	TP + Awiakay
Traditional songs	Awiakay	Awiakay	/
Myths	Awiakay	Awiakay	/ (or just summaries in Awiakay + TP)
Joking	Awiakay	Awiakay	Awiakay + TP

^1^Observations cover three periods: the time of the first author’s first arrival in Kanjimei in 2004, at the start of her PhD fieldwork in 2009 and until 2018 when gold was first discovered, and during her last field-trip in 2025, ca. 5 years into a gold-rush (gold was discovered in 2018, but the gold rush started in 2020/2021)

A series of recent studies of language and ethnobiological skills across PNG has documented intergenerational decline in both TEK and local language proficiency among speakers of numerous languages [29–31]. They concluded that formal education and reduced use /absence of local language in schools are key drivers of this parallel loss. Our study takes a different approach: we examine age- and gender-related variation in TEK and local language use within a single language group – the Awiakay – which is undergoing rapid socio-economic change from a recent gold rush. While the above studies focused on young speakers leaving the community through schooling, we focus on processes unfolding *within* the community.

### Traditional Awiakay subsistence practices

Until recently, the Awiakay subsisted mainly by processing sago, hunting (animals such as wild pigs, cassowaries,^2^ various marsupials, such as possums and bandicoots, bats and birds), fishing (freshwater fish such as catfish, pacu, tilapia, as well as smaller local fishes such as mouth almighty) and gathering (mushrooms, edible leaves and ferns), while supplementing their diet with limited gardening.^3^ Larger reptiles such as crocodiles, monitor lizards and certain types of snakes, pythons in particular, are also killed for food, but they are not deliberately hunted. Fishing is both men’s and women’s work, though the methods differ according to gender. People often emphasise that hunting magic was the domain of men, while fishing magic was the preserve of women. The most popular fishing method, however, is one that involves the whole village. It is called *tiñuŋ tusunpaluŋ* (TP *wasim pis* ‘washing fish’) and is practised at times when the water level in the river and creeks is very low. A few days before the agreed day for ‘washing the creeks’, people go to their gardens to collect *makayp* (the roots of *Derris* sp.), which contains rotenone, a chemical poisonous to fish. The drugged fish come to the surface and swim slowly and more erratically and are thus easier to spear. When the local Konmei creek is ‘washed’, the fish are divided equally between all of the households. As the catch is usually very large, fish are smoked on racks built outside the houses.

In addition to fish, the creeks provide other sources of protein. Young men go diving for turtles and prawns, or try to spear them at night from a canoe. Children also like to catch large grasshoppers that live on the coconut palms, which they cook on a fire and eat as a snack. A highly valued type of food that is usually given to children is the eggs of wild fowl, crocodiles and cassowaries. A variety of insects and plants growing wild in the forest are gathered for food. Apart from various types of fruit (such as *mokos* ‘Malay apples’ (*Syzygium malaccense*; TP *laulau*) or *yambuk* ‘fruits of the plentiful fig’ (*Ficus copiosa*)), gathered food is mainly eaten with sago *tay* (*Metroxylon sagu*). Sago grubs – larvae of sago palm weevils (*Rhyncophorus ferrigineus*; TP *binatang bilong saksak*) called *wao*, are a delicacy. The honey of two types of local stingless bees is also eaten.

Frequently consumed wild plant foods apart from the staple sago, include breadfruit *kamba* (*Artocarpus camansi*; TP *kapiak*) and the yellow and red fruit of various species of pandanus *mañ* (including *Pandanus conoideus*; TP *marita*). Young leaves of various vines and trees, such as *pui* (*Gnetum gnemon*; TP *tulip*) or plentiful fig *yam* (*Ficus copiosa*; TP *kumu mosong*), as well as a variety of edible ferns commonly called *manjom* (including *Diplazium proliferum*; TP *kumu gras*) are also gathered to eat with sago. Among cultivated species, Awiakay have a long history of planting cooking bananas (after clearing small patches of forest), which are often referred to in TP as *kumu bilong tumbuna* ‘vegetables of the ancestors’.^4^ Another crop that grows well and requires little maintenance is sugar cane. As the clay soil in the area is not very fertile, taro and sweet potato were grown with limited success. Garden crops that were introduced very recently (as late as in the 2000s) included pumpkins, cucumbers, beans and corn. The Awiakay usually got the necessary seeds from the neighbouring Asangamut. Plants with edible leaves such as *kolokay* (*Abelmoschus manihot*; TP *aibika*) and a variety of grasses such as lemon grass grow well, and are commonly planted around houses. Betelnut and coconut palms are also planted. A fast-growing plant which is always found in gardens and close to houses is papaya *kanjay* (*Carica papaya*). The Awiakay also grow pineapples, but as they are often destroyed by wild pigs, they have a limited yield.

Awiakay land is dotted with bush camps. Most of these are small clearings in the bush with roughly-built shelters where people stay overnight when they go hunting and processing sago. For about two and a half decades (1995–2020), some of the bush camps were used as base camps for trips to the mountains, where people went to harvest eaglewood (*Gyrinops ledermanii*; TP *garu*), which was highly sought-after by Indonesian buyers (for more on the socio-cultural effects of eaglewood business on the Awiakay see Hoenigman [22].

### The gold rush

The discovery of gold in one Awiakay creek in June 2018 led to a radical shift of community focus towards artisanal mining, and by 2020, the community found themselves in the midst of a gold rush.^5^ This brought a huge influx of outsiders (numbering more than 1,000 individuals), first from neighbouring communities and eventually also from other provinces of PNG, who now regularly travel to Awiakay land as both traders and prospectors. Most Awiakay have largely abandoned communal village life, and now spend long periods of time in gold camps. Artisanal gold extraction by small communities is sometimes discussed in the academic literature in terms of neutral, or occasionally even positive, sociocultural change such as collaboration, inclusion and resistance [38]. More often, however, the focus is on the negative consequences of extraction, such as impoverishment, criminality and environmental degradation [39, 40], language shift away from the indigenous community language [41], the exploitation of community members by outsiders and the breakdown of traditional social norms and structures [42]. The first author’s personal observations suggest that the lifestyle changes that have followed in the wake of the Awiakay gold rush have far more in common with the latter group of studies. The most obvious lifestyle change is the use of money earned by Awiakay gold panners for purchasing the numerous goods brought to the gold camps by traders. Foods such as rice, tinned fish, instant noodles, stock cubes, salt, cooking oil, coffee, sugar, sweets, and soft drinks – as well as alcohol – have become the main diet. By 2023, most people had abandoned their gardens (pers. obs.), the only remaining crops being bananas, sugar cane, tobacco, breadfruit and betelnut. Even sago consumption has declined, largely replaced by the rice brought in by traders. Not only is it more convenient to exchange gold for food immediately after a long day of working in the creeks, but Awiakay women have also become reluctant to pound sago by themselves, because rape has become a real risk in the forest. Yet sago is still seen as “proper food”. People from neighbouring villages – Yamandɨm, Imanmeri, and Ambonwari – have made this their trade by bringing large bags of sago to sell in the gold camps.

As both adults and children, men and women, spend most of their time digging for gold, hunting and foraging are no longer daily activities, but rather an exception in these new circumstances. Sustained contact with outsiders in gold camps has resulted in increased use of TP and a decrease in Awiakay (Table 1). The consequences of this very significant change are starting to show in small children (up to 5 years), who can no longer communicate in Awiakay. While they still know certain Awiakay words, their main language of communication is TP.

### Aims of the study

The central aim of this study was to quantitatively assess the TEK of Awiakay speakers across three age groups. An individual’s ability to name plants and animals in the Awiakay language from stimulus pictures was used as a proxy for his/her level of TEK. In addition, the study investigated potential gender-based differences in the way in which males and females acquire the ethnobiological lexicon. While it is unlikely that a clear quantifiable signal of the disruption caused by the gold rush can already be detected in the data, we present examples of younger individuals, whose TEK has possibly been affected by the aforementioned lifestyle changes. This paper should therefore serve as a baseline against which future Awiakay TEK studies can be compared. Finally, we present a first brief list of culturally relevant Awiakay plant and animal ethnotaxa, with scientific identifications where possible.

## Methods

Considering TEK as part of an overall lifeworld (cf 43, 44), the first author has been combining the methodological approaches of ethnographic enquiry (especially participant observation), linguistic documentation, observational filmmaking and ethnobiology in her fieldwork with the Awiakay. The first author has been working closely with the Awiakay community since 2004, and the observations on Awiakay society, language and culture made throughout this paper are based on this long-term collaboration.

### Study site

Fieldwork for this study was carried out in Kanjimei village between August and November 2025. Awiakay land (Fig. 1) covers approximately 300 square kilometres of lowland rainforest and freshwater swamp forest around the upper Konmei River. This area is in the southern flood plain of the Sepik River in the East Sepik Province of Papua New Guinea. Its southern part stretches into the northern Fringe Highlands of the New Guinea Central Range, and touches the Maramuni River to the south-east. Kanjimei village, where the Awiakay are settled, is located in the north of this territory, at an altitude of 24 m. In the south, the highest mountain on Awiakay land, locally called Injaiŋ, reaches 1311 m.

The Awiakay have been nominal Catholics since the 1950s. They built their first church in 2004. In 2005, they registered Kanjimei Elementary School, and a few generations of children finished the first three grades of elementary school in their home village. Since the beginning of the gold rush, however, there has been no more than a month of tuition each academic year as school children, together with their parents, spend most of their time in gold camps.

Kanjimei has no electricity or running water. There are no roads in the area, and towards the end of the dry season, which lasts from May to September, when the water level in rivers and creeks gets very low, the village is typically accessible only with difficulty by motor canoe. Fallen trees block the river and often need to be cut with an axe in order to pass, and so the journey between Kanjimei and neighbouring Ambonwari may take up to six hours. In the wet season, or when the Konmei fills with water coming from the rain in the mountains, the same distance can be covered in merely two hours. Since the gold rush began, heavy traffic on the river has kept it navigable even during the dry season, as traders and prospectors regularly cut fallen trees and clear blockages.

### Stimulus set

The first author has worked with the Awiakay community since 2004, and has conducted 49 months of fieldwork there, returning regularly over more than two decades. During this time, she has worked closely with local experts, including living with them for extended periods in the forest and learning directly from them about local flora and fauna. This long-term engagement provided an extensive basis for the selection and identification of species included in the study, beyond what would typically be achievable through a pilot study.

**Fig. 2:**
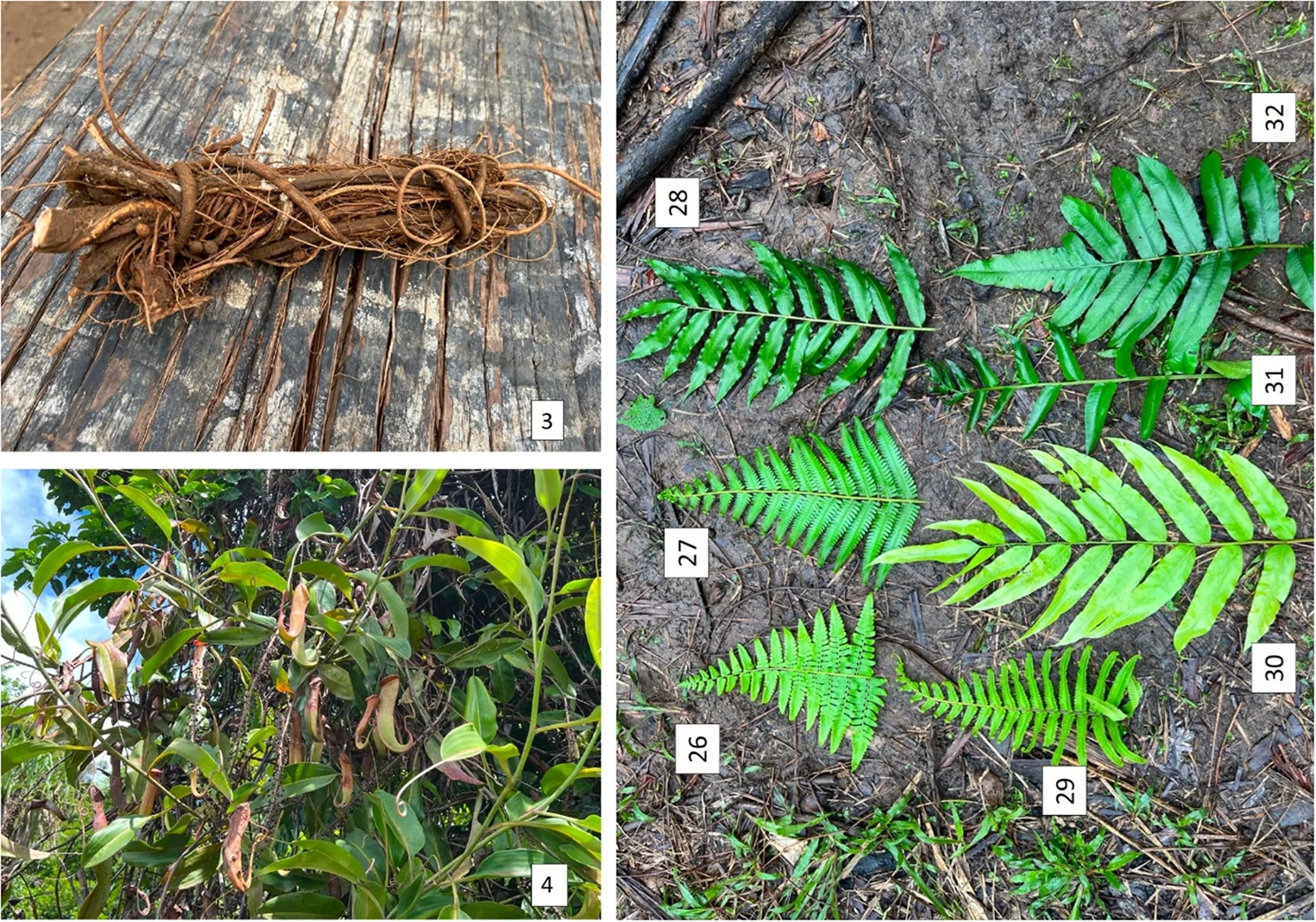
Examples of plant stimuli used in the elicitation task. (3) *Derris* sp., (4) *Nepenthes* sp., (26)–(32) various edible ferns (see bottom of Table 4)

Based on the first author’s experiences, a stimulus set consisting of colour photographs of 23 birds, 25 mammals (including 9 bats) and 32 plants was compiled prior to fieldwork (Fig. 2). The birds were chosen from a species list for East Sepik Province generated by the website Avibase (https://avibase.bsc-eoc.org), whereas the mammals were selected from Flannery and Seri [45]. Some pictures of the mammals had been taken (and their Awiakay names documented) by the first author on previous field trips. All plant photographs had similarly been taken on previous field trips. Occasional duplicate photos of randomly selected species – across all taxa – were included in the stimulus set as an internal quality control for the elicitation procedure. The plant and animal species included in the survey had previously been identified in collaboration with biologists specialising in botany, ornithology and mammalogy (see Acknowledgements for details). While no additional voucher specimens were collected specifically for the present study, the species included had previously undergone expert identification and, in the case of plants, verification through herbarium collections deposited at the PNG National Herbarium [46].

### Participants and elicitation procedure

A total of 62 male and female Awiakay speakers from three age groups were interviewed individually, in the absence of any onlookers. As the first author was living in Kanjimei village itself, potential respondents were simply approached as they became available (i.e., when they were not busy with other tasks). The three age groups were ‘younger’ (8 to 13 years old), ‘intermediate’ (15 to 25 years old) and ‘older’ (40 to 60 years old) (Table 2). Very young (<8 years old) and very old (>60 years old) respondents were not included, as an interview-based elicitation may have proven too taxing for the former, while poor eyesight may have made picture-based identification difficult for the latter. A gap of 15 years was deliberately left between the intermediate and older groups, as we expected the differences in naming ability to be small in adults past the age of 25. In addition, two older males (48 and 59 years old), who were widely acknowledged to be particularly skilled hunters, were interviewed separately. In all, around 12% of the entire Awiakay population was interviewed, although this proportion was far higher for certain age/gender groups (up to 82.4% for the older men; Table 2). If only the targeted age range of 8 to 60 years is considered (351 individuals^6^), the participant pool of *N* = 62 individuals comes to represent 17.7% of the targeted population. The elicitation of plant and animal names was carried out as follows: Respondents were shown pictures from the above-mentioned stimulus set, one at a time, and asked to provide Awiakay names for the plants/animals in the pictures. In some cases (bats, possums, certain plants) they were asked how to distinguish between different types of the same ethnospecies. Respondents would often respond with a TP name, and would have to be prompted to name the organism in question in Awiakay. Responses that were provided in TP-only were also noted. In the case of plants, respondents were also asked about their uses.

**Table 2:** Speaker demographics^1^

	Average Age	N	%
F, younger	11.6	10	33.3
F, intermediate	19.3	9	33.3
F, older	44.8	8	53.3
		Total: 27	
M, younger	12.8	12	36.4
M, intermediate	22.8	9	27.3
M, older	47.1	12	82.4*
		Total: 33	
M, hunters	53.5	2	

^1^The percentages indicate the proportion of individuals from a particular age/gender group who were interviewed. *M (older + hunters)

Finally, a group interview with ten knowledgeable adults (both male and female) was carried out in order to determine the ‘correct’ names of the ethnotaxa in the stimulus pictures through consensus. Seven members of this group had been interviewed separately, but during this final stage, in discussion with others, they changed their minds regarding the names of some ethnotaxa, and accepted names favoured by the majority. The names arising from this group session were the ones used when evaluating the responses in the individual interviews.

### Data analysis

Awiakay speakers’ responses were coded as follows: (1) correct Awiakay name, (2) correct TP name only (i.e. the respondent did not know the Awiakay name) and (3) relevant cultural or ecological knowledge only (i.e. the respondent did not know either the Awiakay or TP name). The proportion of correct responses was averaged for each age group, and separately for males and females. Statistical analyses were carried out on IBM SPSS Statistics Version 31.0. Following tests of normality and homogeneity of variance, ANOVA was carried out to determine the effect of age, gender and a possible interaction of the two variables on respondents’ Awiakay scores. Scores based on TP names or cultural/ecological knowledge only were not subjected to statistical analysis due to the small number of data points.

In a separate analysis, the number of correct responses for each stimulus picture was tallied (separately by age and gender). In this way, the various ethnotaxa represented in the pictures could be graded according to overall familiarity to Awiakay respondents, thereby allowing the identification of lesser-known ethnotaxa or those that are learned with difficulty.

Finally, the younger respondents with the highest and lowest scores were considered separately, with respect to their family background and life history. This allowed a tentative identification of factors that could potentially have affected their TEK. A Spearman’s Rho (ρ) Rank Correlation analysis was also carried out between the variables age and (Awiakay) score for the younger age group, as the scores were not normally distributed. Mann-Whitney U Tests were carried out to compare children who had significant exposure to mining versus those who did not. Comparisons were made separately for boys and girls.

## Results

### Statistical analysis

**Fig. 3:**
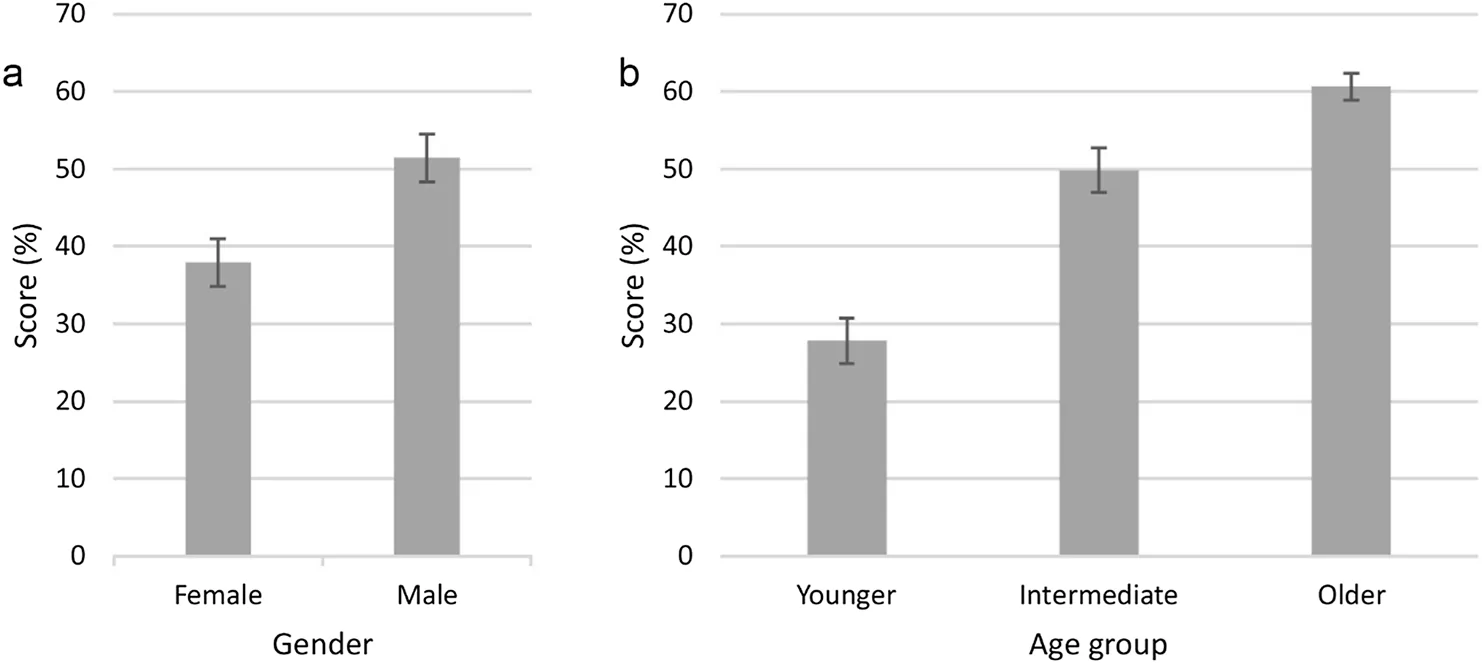
Responses (Awiakay names only) pooled by (**a**) gender and (**b**) age. Error bars show Standard Error.

The ANOVA revealed that, overall, male respondents were able to provide more Awiakay names for the ethnotaxa in the stimulus pictures than female respondents (Fig. 3a, F = 28.19, *p* < 0.001). There was also a significant effect of age on the number of Awiakay names provided (Fig. 3b, F = 65.03, *p* < 0.001), with older respondents being able to name the most ethnotaxa. There was no significant interaction of age and gender (F = 1.97, *p* > 0.05).

**Fig. 4:**
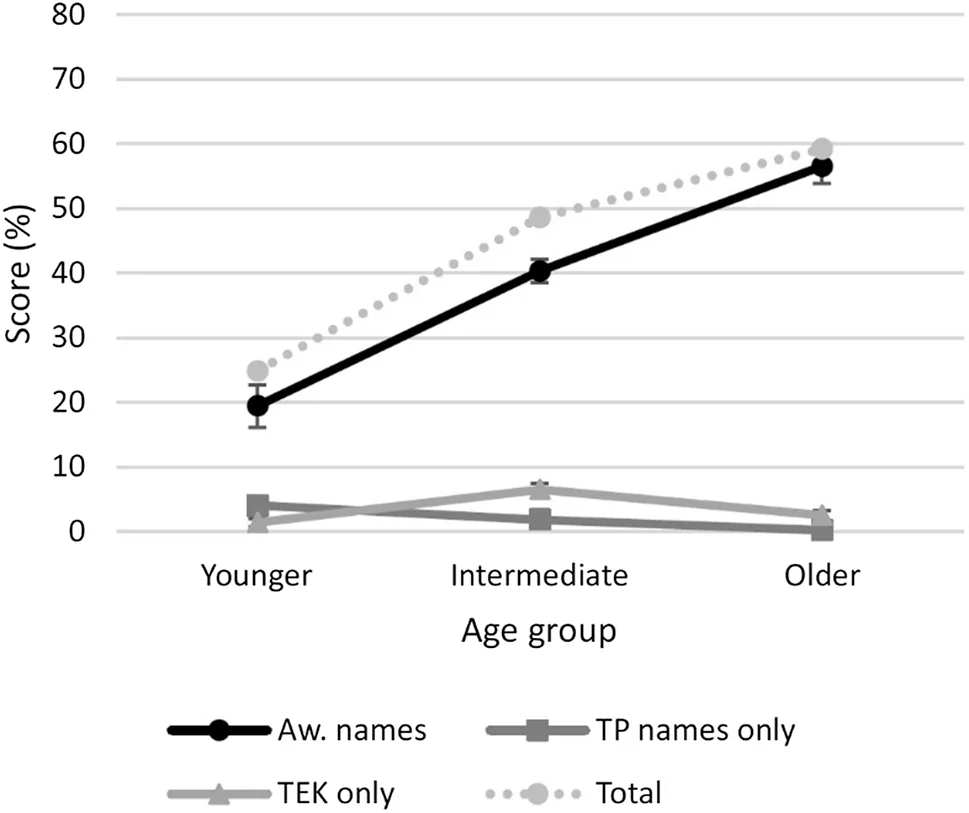
Combined plant and animal knowledge, across all taxa, among female respondents. The solid lines show stimuli identified through (**a**) Awiakay names, (**b**) TP names only and (**c**) not identified through a lexical label but through relevant TEK (cultural, ecological knowledge). The dotted line shows the sum of all three indices

**Fig. 5:**
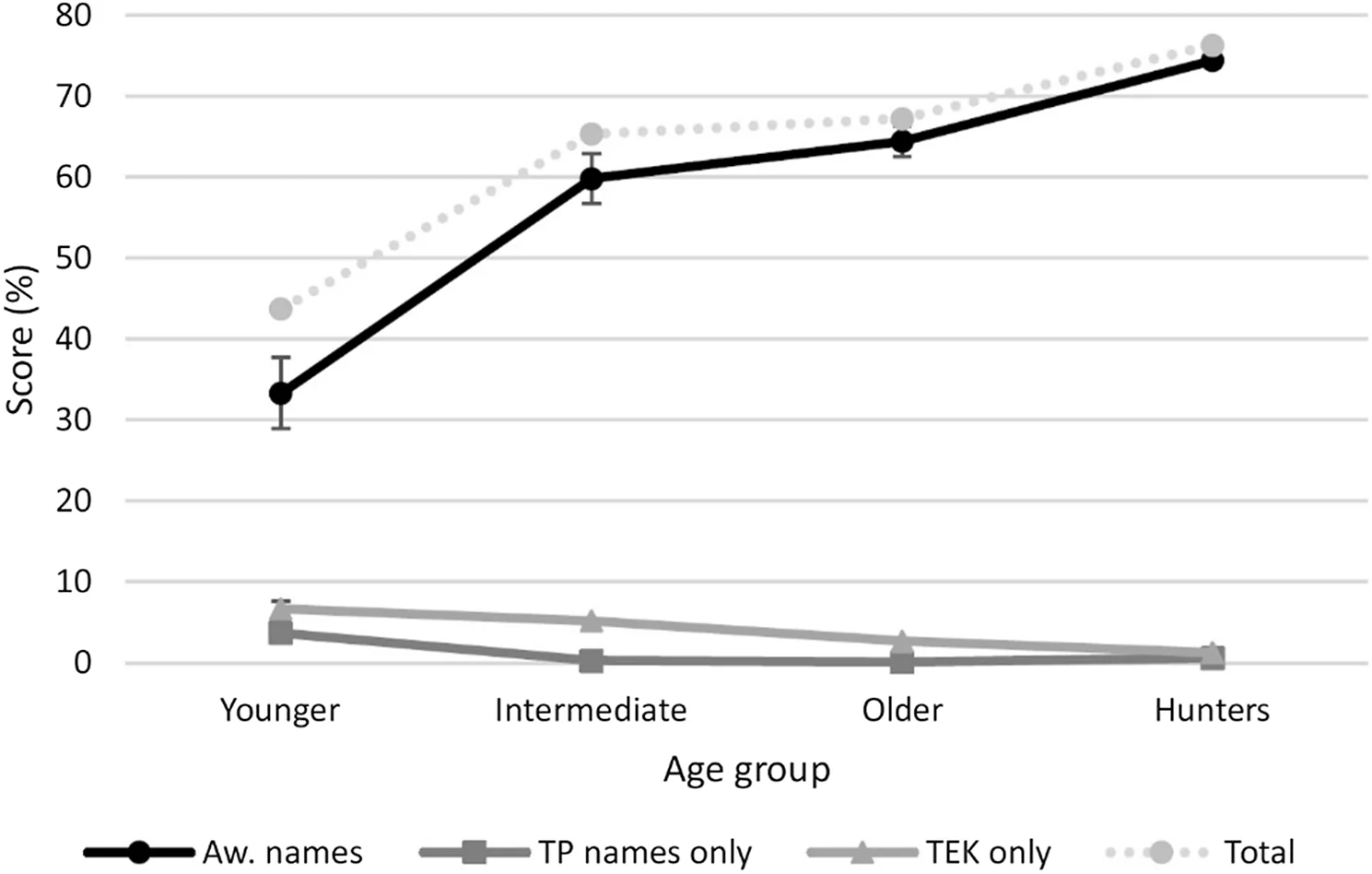
Combined plant and animal knowledge, across all taxa, among male respondents (including two expert hunters, shown separately). The solid lines show stimuli identified through (**a**) Awiakay names, (**b**) TP names only and (**c**) not identified through a lexical label but through relevant TEK (cultural, ecological knowledge). The dotted line shows the sum of all three indices

Despite the lack of a significant age-gender interaction in the overall scores, the patterns of Awiakay lexical acquisition for males and females do seem to be different (Figs. 4 and 5). Among female respondents, acquisition seems to be a linear process that continues steadily until middle age (~45 years), whereas among males, knowledge of Awiakay plant and animal names seems to have plateaued by early adulthood (~20 years). The only improvement in scores past this point was seen among the skilled hunters; however, no statistical analysis was carried out, because the latter category contained only two data points. It is also noteworthy that males outperformed females even within the younger age group (*t* = −2.47, d.f.=21, *p* < 0.05).

Some responses consisted of only TP names, or only relevant cultural or ecological knowledge (Figs. 4 and 5). In the latter type of response, the Awiakay name was not known to the respondent. The addition of such responses to the Awiakay data did not change the overall age- or gender-related patterns. Examples include:

*aŋgiaŋ: *the flowering vine* Freycinetia marginata. *Almost all respondents knew what it looks like and where one could find it, but only one knew its Awiakay name.

*wauna: Nepenthes *sp., TP kap. All respondents knew that the pitcher of this vine is used as a* kap ‘*cup’ in which one can collect water and drink from. However, it was only adults who knew its Awiakay name.

*kaŋgam: *the Lesser Bird of Paradise* (Paradisaea minor), *TP* kumul.* Most children (77%) and a few of the intermediate group (33%) only knew the TP name.

*kiandok:* Brown’s Pademelon* (Thylogale browni), *TP *sikau. *Young boys sometimes used the TP name while girls did not recognise it at all.

The proportion of correct TP-only responses was the highest in the younger group. Figures 4 and 5 show the percentage of stimulus pictures that elicited TP-only responses. This was quite low, with mean values of less than 5% (of all responses – correct and incorrect) for both boys and girls. However, the number of correct responses for the younger age group was also quite low, and when the TP-only percentage is recalculated as a proportion of correct responses only in either language, the figures are higher: 9% for boys and 15.7% for girls. Children, especially girls, had trouble identifying bats. They either used a TP term *plain poks* (flying fox) ~ *bilak bokis* or used the Awiakay word *ayŋgwaŋ* as if it were a generic term. However, adults insisted that there is no generic term for bats in Awiakay and that *ayŋgwaŋ* only refers to Bismarck flying fox *(Pteropus neohibernicus).*

**Fig. 6:**
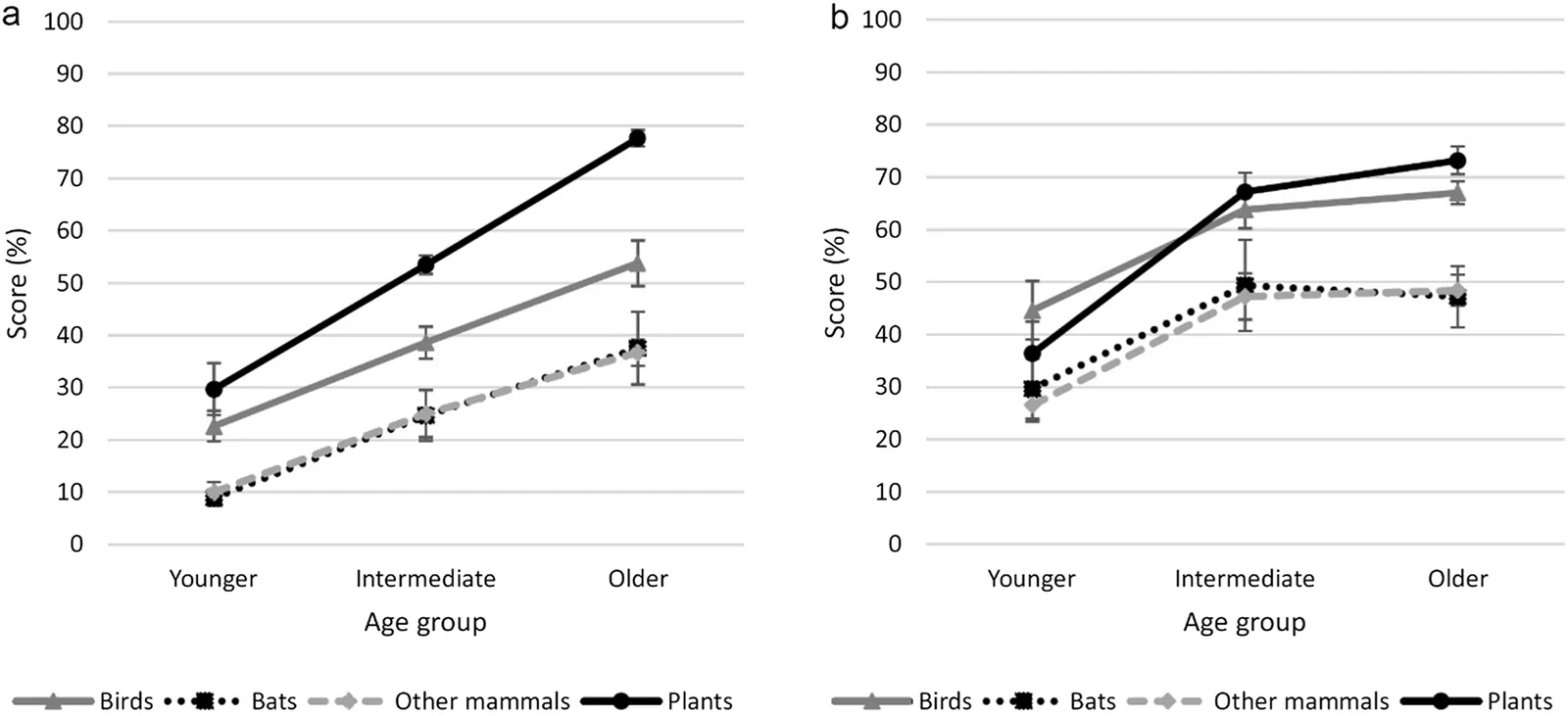
Results of the naming task for each taxon separately (left: females, right: males). The percentages indicate the proportion of plant and animal stimuli for which correct Awiakay names were provided. The results do not include responses consisting of only the TP name or only relevant cultural/ecological knowledge

Analysis of the respondent scores (Awiakay only) by taxon revealed largely similar trends for both females and males. In general, both had greater knowledge of the names of plants and birds than of bats and other mammals (Fig. 6). The same pattern could be seen in the hunters’ responses: plants 90.6%, birds 71.7%, other mammals 59.4%, bats 50% (not shown in Fig. 6). In the case of the female respondents, the linear increase in Awiakay ethnobiological vocabulary noted above can also be seen for each taxon separately (Fig. 6a).

### Familiarity of ethnospecies

The Awiakay and TP names of the ethnospecies represented in the stimulus sets are shown in Tables 3 and 4. Also shown are the ability of the adult (intermediate and older age groups combined) and child (younger age group) respondents to name each ethnospecies. Note that *a* and a* represent the highest number of correct responses in Awiakay, whereas *c* and c* represent the lowest. There were some ethnospecies (such as the Victoria Crowned Pigeon, the Common Spiny Bandicoot or the Ringworm Bush) that were easily recognised and named by all respondents, regardless of age or gender. However, gender-based differences were apparent in the recognizability of some ethnospecies; these included the Azure Kingfisher, which was much better-known among the adult male respondents, and the Zoe Imperial Pigeon, which was recognized by over two-thirds of the men and boys, but by less than one-third of the girls.

**Table 3:** Animals included in the visual stimuli (birds, bats, other mammals), their local names and naming ability for each stimulus among female and male respondents

Common name	Scientific name	Local name(s)	F, M	f, m
*Birds*				
Twelve-wired Bird-of-Paradise	*Seleucidis melanoleucus*	kaŋganam (male); muŋuyn (female)	c, c	c*, c
Blyth’s Hornbill	*Rhyticeros plicatus*	kyakwi ~ kaykwi; TP kokomo	a*, a	b, b
Azure Kingfisher	*Ceyx azureus*	yumuñ ~ yomoñ; TP longpela nus	c, a	c*, b
Papuan Frogmouth (1)	*Podargus papuensis*	komam	b, a	c, b
Australasian Darter	*Anhinga novaehollandiae*	miŋ	c, b	c*, b
Common Paradise-Kingfisher	*Tanysiptera galatea*	oloŋ	c*, c*	c*, c*
Black Kite	*Milvus migrans*	naim; TP tarangau	a, a	b, a
Brahminy Kite	*Haliastur indus*	wanjikay [naim]; TP tarangau	b, a	c, b
Dollarbird	*Eurystomus orientalis*	takañ ~ takayn	c, b	c, b
Great-billed Heron	*Ardea sumatrana*	wiginam ~ wikinam ~ uyginam	c, b	c, b
Lesser Bird-of-Paradise	*Paradisaea minor*	kaŋgam; TP kumul	b, b	b, b
Edwards Fig Parrot	*Psittaculirostris edwardsii*	sekek	c, b	c*, b
Intermediate Egret	*Ardea intermedia*	kakoy; TP saun	b, a	c, b
New Guinea Flightless Rail	*Megacrex inepta*	wanday imbiambam amgoy^1^	c*, c	c*, c*
New Guinea Scrubfowl	*Megapodius decollatus*	naŋgao; TP (liklik) buspaul ~ paul bilong bus	c, c	c*, c
Palm Cockatoo	*Probosciger aterrimus*	aŋgiñaŋ; TP blek koki	b, a	c, a
Papuan Frogmouth (2)	*P. papuensis*	komam	a, a	b, b
Pygmy Parrot	*Micropsitta* sp.	kinjain^2^	c, c	c, c*
Red-legged Brushturkey	*Talegalla jobiensis*	pandombao; TP wail paul ~ wail kakaruk	c, b	c*, c
Sulphur-crested Cockatoo	*Cacatua galerita*	aiwa; TP (wait) koki	a*, a*	a, a
Western Corella	*Cacatua pastinator*	aiwa; TP (wait) koki	c, b	c, b
Zoe Imperial Pigeon	*Ducula zoeae*	opum; TP balus	b, a	c, a
Victoria Crowned Pigeon	*Goura victoria*	ambay	a*, a*	a, a*
*Bats*				
Bismarck Flying Fox	*Pteropus neohibernicus*	ayŋgwaŋ ~ ayngan^3^; TP bilak bokis ~ plain poks	a, a	a, a
Diadem Roundleaf Bat	*Hipposideros diadema*	waypa	c, c	c*, c
Dark Sheath Tailed Bat	*Mosia nigrescens*	apuŋ	c, b	c*, b
Geoffroy’s Rousette	*Rousettus amplexicaudatus*	kondomom	c, c	c*, c
Southern Blossom Bat	*Syconycteris australis*	waypa	c*, c	c*, c
tube-nosed fruit bat (1)	*Nyctimene* sp.	waypa	c, b	c*, c
?Bare-backed Fruit Bat	*Dobsonia moluccensis*	kondomom	c, c	c*, c*
tube-nosed fruit bat (2)	*Nyctimene* sp.	waypa	c, c	c*, c
?Papuan Pipistrelle	*Pipistrellus papuanus*	apuŋ	c, b	c*, b
*Other mammals*				
Black Spotted Cuscus	*Spilocuscus rufoniger*	mi tapuŋga [pawia meŋgea]; TP kapul (generic name)	c*, c*	c*, c*
Common Lowland Paramelomys	*Paramelomys platyops*	kunduk; TP rat (bilong bus)	b, a	a, a
Common Spiny Bandicoot	*Echymipera kalubu*	andaŋgay [kamao]; TP mumut	a, a*	a, a*
Brown’s Pademelon (1)	*Thylogale browni*	amanjok [kiandok]; TP sikau (bilong maunten)	b, b	c*, c
Brown’s Pademelon (2)	*T. browni*	amanjok [kiandok]; TP sikau (bilong maunten)	c, c	c*, c
Ground Cuscus (1)	*Phalanger gymnotis*	kumaka [pawia meŋgea]; TP blekpela kapul	b, a	b, b
Common Spotted Cuscus (1)	*Spilocuscus maculatus*	kiaŋgay [pawia meŋgea]	c, a	c*, c
Feathertail Possum	*Distoechurus pennatus*	kamao pawa	c*, c*	c*, c*
Ground Cuscus (2)	*P. gymnotis*	kumaka tenja [pawia meŋgea]	c, b	c*, c
Lowland Ringtail Possum	*Pseudochirulus canescens*	yaŋgok kumaka [pawia meŋgea]	c, b	c*, b
Common Spotted Cuscus (2)	*S. maculatus*	kiaŋgay [pawia meŋgea]	c, b	c*, c
New Guinean Quoll	*Dasyurus albopunctatus*	pandambim^4^	c*, c*	c*, c*
Northern Common Cuscus	*Phalanger orientalis*	yaŋgok kumaka [pawia meŋgea]	b, b	c*, b
Short-furred Dasyure	*Murexia longicaudata*	kuŋgwi	c*, c	c*, c*
Sugar Glider	*Petaurus breviceps*	semek	c, c	c*, c*
Water Rat	*Hydromys chrysogaster*	taymboyn	c, b	c*, c

(1), (2) repeated stimulus picture; Local name(s) column shows Awiakay name, ~ indicates phonological/lexical variation, superordinate term in square brackets, TP Tok Pisin name (if known); columns F and M indicate overall frequency with which adult female and male respondents (‘intermediate’ and ‘older’) provided Awiakay names for each stimulus; f and m indicate the same for ‘younger’ respondents only: a*: all respondents, a: >2/3 of respondents (but not all), b: >1/3 and up to 2/3 of respondents, c: ≤1/3 of respondents, c*: 0 respondents

^1^*wanday imbiambam amgoy* literally means ‘chicken of the bush’, but it can also just be called *wanday*. The speakers added the descriptive term to distinguish it from domestic chicken, also called *wanday*

^2^While there was a general consensus that this bird is *kinjain*, several people called it *mimbikiñ*, which had been previously identified as Pygmy Parrot

^3^*ayngan* is a variant form used by some of the younger generation

^4^This animal is rarely seen on Awiakay land and many respondents said that they did not know it. In the final discussion, consensus was reached that it lives in the mountains

**Table 4:** Plants included in the visual stimuli, their local names and naming ability for each stimulus among female and male respondents

Common name/ Description	Scientific name	Local name(s)	F, M	f, m
Golden Bamboo	*Bambusa vulgaris* var. *striata*	mekay; TP mambu	b, b	c, c
Branched comb fern	*?Schizaea dichotoma*	yomblay kambanjagay ~ kao imba namonum imba	c, c	c*, c
Fish poison vine	*Derris* sp.	makayp	a*, a*	a*, a*
Pitcher plant	*Nepenthes* sp.	wauna	c, c	c*, c
Liana (fruit) (1)	*Trichosanthes schlechteri*	wapukam	b, a	c, c
Liana (leaves) (2)	*T. schlechteri*	wapukam imba ‘vine’	c, b	c*, c
Leichhardt Tree (tree) (1)	*Nauclea orientalis*	karoŋ (isa ‘tree/wood’)	b, b	c, c
Leichhardt Tree (fruits) (2)	*N. orientalis*	karoŋ (maŋga ‘fruit’)	c, b	c, c
Leichhardt Tree (flowers) (3)	*N. orientalis*	karoŋ (waia ‘flower’)	c, b	c, c
Ant tree (1)	*Endospermum moluccanum*	pioŋ (kaŋga ‘leaf’) ~ piauŋ	b, b	c, c
Ant tree (2)	*E. moluccanum*	pioŋ (isa ‘tree’) ~ piauŋ	b, a	c, c
Fishtail Palm	*Caryota rumphiana*	kuplay; TP limbum	b, a	c, b
Nettle	*Laportea decumana*	mamay; TP salat	a*, a*	a, a
Pandanus fruit	*Pandanus conoideus*	wakoñ	a, a*	a, a
Ringworm Bush	*Cassia alata*	kisakisak	a*, a*	a*, a
Flowering vine	*Freycinetia marginata*	aŋgiaŋ	c*, c	c*, c*
Floating plant	*Hanguana anthelminthica*	koŋgonoŋ	b, a	c, b
Smooth Ant Plant	*Hydnophytum moseleyanum*	payp	c, c	c*, c*
Vine	*Monstera* sp.	kumum	c, b	c, c
Sago	*Metroxylon sagu*	mongoy tay	b, b	b, b
Turmeric	*Curcuma longa*	wari; TP tumerik	c, c	c*, c*
Forked fern	*?Dicranopteris linearis*	yomblay	c, b	c, c
Pandanus	*Pandanus* sp.	yupi (bush variety)^1^	c, c	c*, c
Veiled Lady Mushroom	*Phallus indusiatus*	tam yoŋga ‘dog penis’ ~ tam tukumba ‘dog testicles’	b, b	c, c
Fungus on wood	?	yambiyam kuma ~ kaiŋ ~ is mania	b, b	c*, c
Edible fern a.	*Diplazium esculentum*	manjom	b, b	b, b
Edible fern b.	*Diplazium* sp.	muñelek	a, b	a, c
Edible fern c.	*Stenochlaena* sp.	kiñigam ~ kingam ~ tinjikam	b, b	b, c
Edible fern d.	*Diplazium dilatatum*	wambi (manjom kumuña)	b, c	c, c
Edible fern e.	*Stenochlaena* sp.	tendem	a*, a*	a, a
Edible fern f. (1)	*Diplazium* sp.	ambam (tukusa ‘young leaves’)	a, a	b, b
Edible fern f. (2)	*Diplazium* sp.	ambam (kawia ‘old leaves’)	a, a	a, b

(1), (2) repeated stimulus picture; Local name(s) column shows Awiakay name, ~ indicates phonological/lexical variation, TP Tok Pisin name (if known); columns F and M indicate overall frequency with which adult female and male respondents (‘intermediate’ and ‘older’ combined) provided Awiakay names for each stimulus; f and m indicate the same for ‘younger’ respondents only: a*: all respondents, a: >2/3 of respondents (but not all), b: >1/3 and up to 2/3 of respondents, c: ≤1/3 of respondents, c*: 0 respondents

^1^The swamp variety of this pandanus is called *kiakay*

### TEK and life history: younger respondents

The effects of the gold rush in Kanjimei on TEK, if any, are most likely to be observed in the responses of the younger Awiakay speakers. Children who were 10 years old or younger at the time of interview would have been toddlers at the time the gold rush started, and therefore in a sensitive phase for the acquisition of TEK and language between that time and the time of interview.

**Fig. 7:**
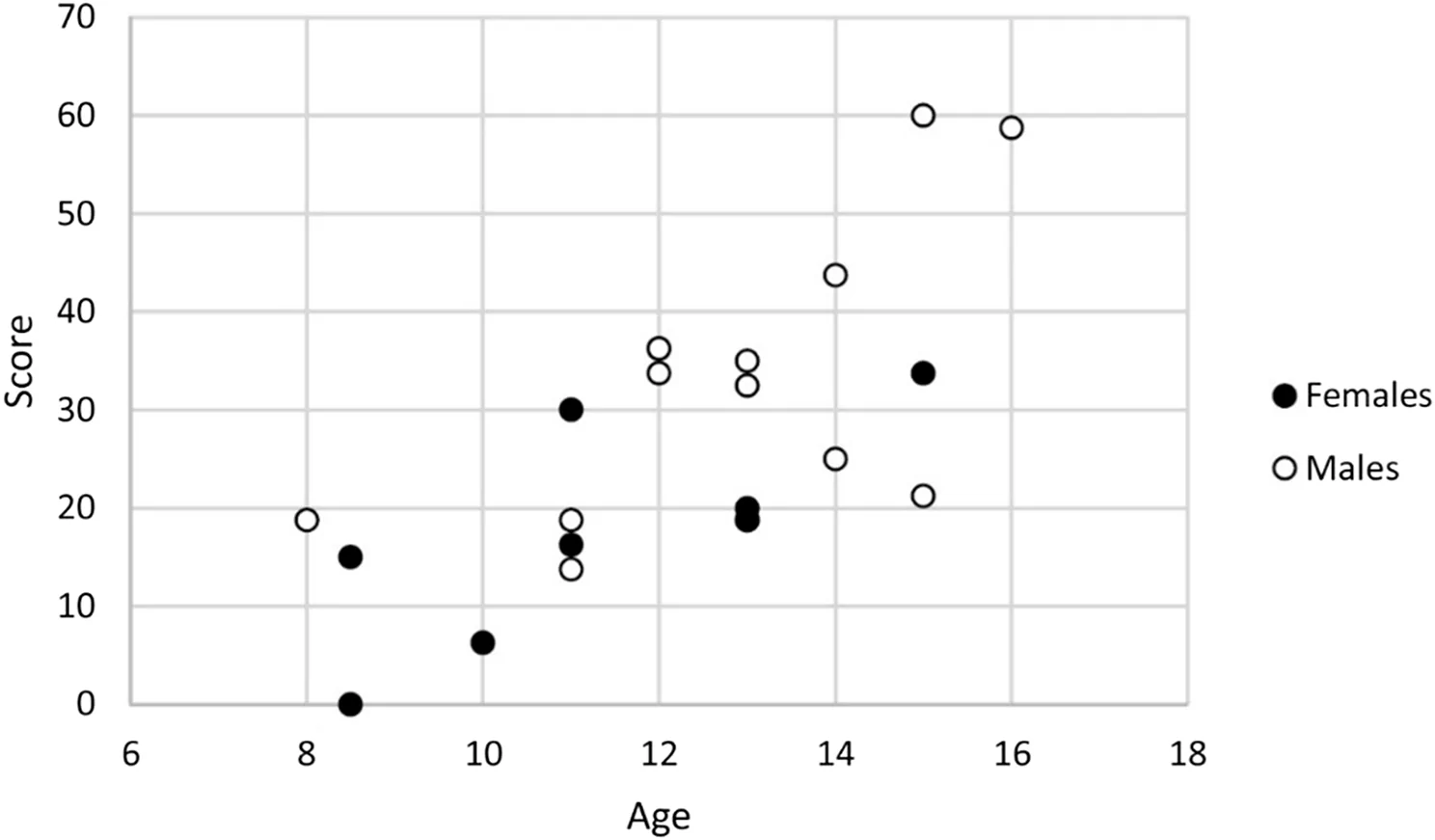
Change in Awiakay responses with age (younger group only)

There was a positive, statistically significant correlation between age and interview score (Fig. 7) for both boys (Spearman’s Rho (ρ) Rank Correlation, (ρ = 0.675, *p* = 0.023)) and girls (Spearman’s Rho (ρ) Rank Correlation, (ρ = 0.670, *p* = 0.017)). It is possible that this represents, at least partly, a normal TEK acquisition curve for children, and it cannot be concluded, from the interview scores alone, that the low-scoring children had experienced some kind of disruption in TEK transmission due to the gold rush.

**Fig. 8:**
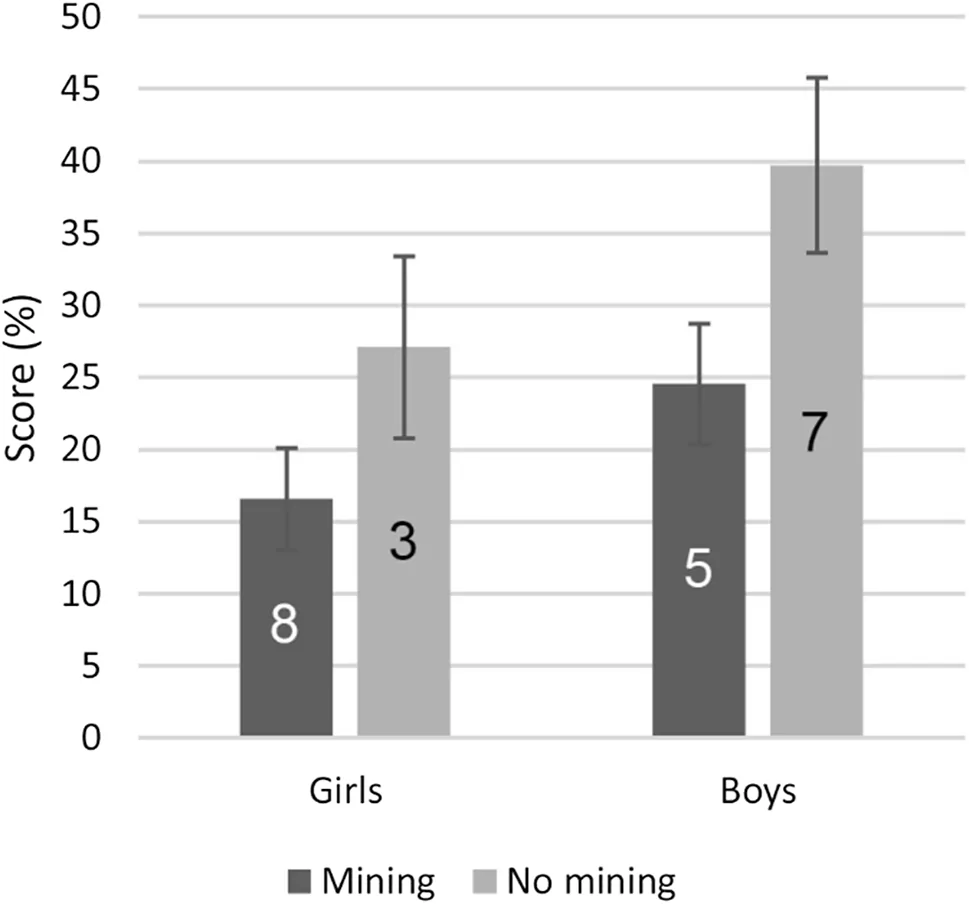
Mean scores of the children from Fig. 7, grouped according to their exposure to mining. Error bars indicate standard error, and the number within each column indicates the number of children in that category

Comparisons of boys and girls with significant or little exposure to mining showed a tendency for the latter group to have higher scores (Fig. 8), but the difference between the two groups was not statistically significant for either gender (Mann Whitney U Test – girls, *U* = 7, *p* = 0.38; boys, *U* = 7, *p* = 0.09).

In the following, we focus on the life histories and family backgrounds of individual boys and girls who achieved the highest and lowest scores (in terms of being able to name plants and animals in Awiakay). The life history/family information, combined with the interview scores, should bring to light any adverse effects of the gold rush – or any other disruptors – on TEK transmission. The interviewees have been assigned anonymised codes. The ‘Awiakay score’ shown after the code and the child’s age indicates his/her raw score (i.e. total number of stimuli correctly identified and named with an Awiakay label), along with the percentage of stimuli named correctly by that child.

#### Girls with low scores

F1: 8.5 years old [Awiakay score: 0 (0%)] Her result was extremely poor, even for her young age. She did not know a single Awiakay name and was only able to produce one bird name in TP. Her family (both parents and her two older siblings) have been spending extended periods of time in gold camps ever since she was 2 years old. She was therefore not following her mother(s) on foraging trips like her peers, whose parents spent more time in the village, leading a more traditional lifestyle.

F2: 10 years old [Awiakay score: 5 (6.3%)] She was 3 years old when her mother became paralysed. Her father has since been spending long periods of time in gold camps, away from his family, where he became an alcoholic. Even when he returns to the family in the village, he is not motivated to teach her about TEK. Although she sometimes spends time with her classificatory mothers, she has had less opportunity to join adults on foraging trips than her peers.

F3: 11 years old [Awiakay score: 13 (16.3%)] She was 5 years old when her parents started spending extensive periods of time in gold camps. While they are both very knowledgeable about the environment, panning for gold does not allow much opportunity for hunting and foraging.

#### Boys with low scores

M1: 11 years old [Awiakay score: 11 (13.8%)] As with his younger sister, F1, he achieved the lowest score among the boys. Note that compared to his sister, his result is considerably better, which is to be expected, given that he is almost two years older. His family has been spending extended periods of time in gold camps ever since he was 4 years old. Like his sister, he was not following his mother(s) on foraging trips, nor did he go hunting or setting traps with his father.

M2: 8 years old [Awiakay score: 15 (18.8%)] He was the youngest of all respondents, and this probably explains his lower score. His family are very knowledgeable about the natural environment and are proactive in educating their children about it (his father is also Author 1’s main research assistant). Consistent with others in his age group, he provided several plant and animal names in TP (25% of his correct answers were in Tok Pisin) rather than in Awiakay. Note that even though his score is among the lowest of the males of the younger group, he did considerably better than F1 (above), who is of a similar age. His family spend far less time in the gold camps than most other villagers.

M3: 11 years old [Awiakay score: 15 (18.8%)] He was just 4 years old when gold was discovered, and, together with his parents, spent a major part of the next few years in gold camps, with only short periods of time spent in the village. In 2022, his elder brother contracted a chronic infection, and the family moved back to Kanjimei. However, with sickness in the family, people do not tend to go on longer bush trips, so throughout his formative years, M3 had fewer opportunities to learn TEK in situ, despite the fact that his parents and grandparents are very knowledgeable about the environment, and the male members of the family are regarded as good hunters.

#### Girls with high scores

F4: 11 years old [Awiakay score: 24 (30%)] She and her three siblings are the only children in the village who are being raised by a single mother, who has never been married to any of her children’s fathers. Not having an adult male in the family, they all have to spend more time finding food for themselves, and thus spend more time in the forest.^7^ While they do spend periods of time in gold camps, not having an adult male in the family also means that their yield is lower than that of others, and they cannot fully depend on gold for purchasing food. The family thus tends to divide their time more equally between the gold camps and the village, where they mostly subsist on fishing and foraging.

F5: 12 years old [Awiakay score: 29 (36.3%)] She was 5 years old when gold was discovered, but her parents did not immediately rush to the gold camps. Although they did spend periods of time there later on, they have never abandoned the village for more than a couple of months at a time.

F6: 15 years old [Awiakay score: 27 (33.8%)] She was 8 years old when gold was discovered, and although her parents later practically moved to one of the gold camps, it was not until she was 11 or 12 years old. Thus, she spent her formative years foraging with her older siblings and female relatives, as was the case with previous generations.

#### Boys with high scores

M4: 15 years old [Awiakay score: 48 (61.3%)] His family have never spent much time in gold camps. Both his parents are very knowledgeable about the environment, and his father is a very good hunter. In addition, they have worked closely with Author 1 since before M4 was born, so he too became used to travelling with her in the bush and hearing his parents and his older siblings passing on TEK to her.

M5: 16 years old [Awiakay score: 47 (58.8%)] He was 3 years old when his mother died of a snakebite. He was raised by his widower father and his elder sister, and the latter has been taking him to the bush with their classificatory mothers and sisters. His father did not immediately rush to the gold camps when the gold was discovered, and so M5-16 was exposed to TEK during his early formative years.

M6: 15 years old [Awiakay score: 39 (48.8%)] His result was a bit of a surprise, given that his mother is from a faraway village and communicates mostly in Tok Pisin, although she now has receptive knowledge of Awiakay. However, much like the majority of the village, M6’s family have always lived a very ordinary lifestyle consisting of daily trips to the bush. In addition, they did not rush to gold camps as soon as gold was discovered, which contributed to his acquisition of TEK in those crucial years.

## Discussion

The above results can be summarised as follows: overall, males performed better than females in the naming task, but the acquisition of the ethnobiological lexicon over the lifetime seems to vary between the two sexes. Women seem to acquire Awiakay plant and animal names steadily well into adulthood, whereas men seem to have acquired most of the relevant vocabulary by their early 20s. Both groups performed better with plant and bird names than with mammal names, and it was only in the ethnobotanical domain that the older women’s performance matched, or even superseded, that of their male counterparts. Children of both sexes whose parents were highly involved with the gold rush tended to have the lowest scores; however, boys and girls also had very similar acquisition curves, which indicates that any disruption in TEK transmission caused by the gold rush is likely affecting both to a similar degree. In the following sections, we discuss three of our main findings in more detail. These are age and gender effects on TEK, language shift to TP and the impact of the gold rush. Much of the ethnographic information presented below is from the personal observations and field notes of the first author, and has not been previously published.

### Age

Numerous studies have demonstrated an effect of consultant age on ethnobiological knowledge (e.g. 48), with the general pattern being, as in the current study, that knowledge increases with age. However, a further noteworthy observation in the literature is that knowledge acquisition need not be gradual. Young adults may already have considerable knowledge in specialist occupations such as fishing [49] and even children frequently demonstrate adult-like general knowledge of useful plants [50–52]. In the current study, the performance of the younger respondents was significantly lower than that of the intermediate group (i.e. young adults), but a closer look at the individual scores of the younger respondents shows a tendency towards young-adult like knowledge levels. This is particularly true of three of the male teenagers (Fig. 7), whose scores in Awiakay ranged between 40%-60%, and is comparable to the performance of the intermediate (adult) group.

Overall, however, the children in the present study who were younger than 12 years old did not show the ‘precocious’ naming abilities observed by Hunn [50] and Zarger [51] for children of a comparable age. One possible reason is the nature of the stimuli presented to the respondents. First, naming plants and animals from photographs is more difficult than identifying them in their natural habitat [53, 54], because of a ‘decontextualization from the landscape or the season’ [48]. Had the children been allowed to wander freely through the environment, and collect and name plants known to them (as was the case with Hunn [50]), they may have been able to demonstrate a higher level of TEK. Second, the stimuli used in the present study contained wild plants and animals exclusively, and not the household garden plants, which were the subject of the Zarger [51] study. The inclusion of wild plants, birds and mammals – the bats in particular –made the task more challenging, and the overall scores would have probably been higher if the stimulus set had only contained well-known edible plants. However, these constraints apply to all the consultants interviewed in the present study, and we believe that the age- and gender-based differences evident in the results are valid. Wild plant and animals are also more culturally appropriate for the Awiakay because, as mentioned in the Introduction, the community have relied heavily on wild foods to complement their garden produce (27: 102–104).

In general, the Awiakay teach their children about the environment in situ, and through lived experience. This is very much in line with the observations made by Ingold [55] and Lancy [56], who stress that being present in the natural environment, and having repeated interactions with plants and animals – either through work or play – are crucial for the acquisition of TEK. The first author of the current paper has heard a father calling his son back from playing in the high grass after nightfall and explaining how various animals come out at night to hunt, and how staying there could result in being bitten by a snake. Prior to the gold rush, young Awiakay children would accompany their mothers on daily foraging trips, during which they gradually acquired knowledge of the most commonly gathered plants, including edible ferns, tree leaves, and mushrooms.

Boys would soon become more independent. They would spend time with older siblings and peers, making their own small bows and arrows with which to hunt lizards, frogs, rats, and even birds. They would also collect grasshoppers from coconut palms in the village, and go spearing prawns and fish at night. These were mostly eaten as snacks, cooked over a fire and shared with their peers. However, once young teenage boys learned to spear fish and kill small mammals such as bandicoots, they would start bringing their catch back to their families. At this stage, they would also occasionally accompany their fathers to set traps for larger game such as pigs, cassowaries and Goura Pigeons, or go on night hunting trips with their older brothers. Meanwhile, girls would remain with their mothers, looking after younger siblings, and helping with foraging, fishing, fetching water, breaking firewood, and cooking.

In a society where hunting and gathering structured daily life, references to TEK were embedded in every aspect of social and cultural practice. In the time when the Awiakay still practised initiations, both male and female initiation rituals were tightly intertwined with deep environmental knowledge. The female initiation ritual, for example, was a detailed re-enactment of female hornbill nesting practices, whereby the female hornbill seals herself into a tree hole during incubation, and is fed by a male through a small opening [57]. Hornbill behaviour is still used to explain both male and female rituals, just as human kinship is used to explain hornbill behaviour.

Environmental knowledge is also reflected in the Awiakay practice of making string figures [58]. Of 71 known patterns, 30 represent animals, 8 represent plants, and 4 depict elements, landscape features, or natural phenomena. Among the figures representing people and their activities, 3 depict activities connected with plants and animals. Thus, more than half of all string figures are directly connected with TEK. In some cases, children’s make-believe, in the context of some string figures, reflects knowledge of animal behaviour. For example, the way rats come to steal food at night is enacted in play through string figures. In the case of the figure called *kamao* ‘bandicoot’, the final design resembles the pointed nose of a bandicoot (*Echymipera rufescens*). The figure-maker then goes on to make the bandicoot’s stick nest. The animal now needs to be killed, so the maker cracks their knuckles, imitating the sound of breaking the bandicoot’s neck. In the figure called *kambam* ‘catfish’, when the design emerges, the maker says they will “get rid of the side bones” and slides the figure along their thigh, demonstrating how easily the bones are removed from this fish. In a similar way, each string figure encodes detailed ecological knowledge.

Although the Awiakay seldom explicitly teach children about plants and animals, activities such as string-figure making serve as an indirect means of transmitting environmental knowledge, and can prompt direct explanation. The first author once witnessed a man watching his children make the bandicoot figure, and then begin explaining how bandicoots are trapped and killed. As with many cultural practices, however, string-figure making is also being abandoned for modern pursuits (such as watching TikTok videos), and nowadays even the most skilled string-figure makers no longer have the inclination to indulge in this traditional activity.

### Gender

The gender differences found in the present study can be attributed to the fact that, in general, women do not hunt; this seems to have resulted in a reduced ability to identify and name certain birds and mammals, particularly in the younger and intermediate age groups. Other studies have also reported such a pattern, with the consensus being that women specialise in the collection of plant material (while hunting is left to the men), and are therefore more knowledgeable about ethnobotanical issues [59, 60]. However, this need not be the case everywhere (61; 62): 215–217 63; see Pfeiffer & Butz [24] for a review), and it has been noted that in many communities around the world, men are ‘jacks-of-all-trades’, and are often equally knowledgeable about plants as women [25]. This can be seen in the data presented above, where older men and women performed equally well (70%-80% correct plant responses) in the plant-naming section of the interviews. Indeed, the two (male) expert hunters, whose data were analysed separately, attained scores of 84 and 97%. Souto and Ticktin [14] also observed similar levels of knowledge of useful plants among older men and women in mestizo communities in Venezuela.

Being closest in their size to man, cassowary and pig are big game animals who are ascribed the most human characteristics, and hunting either of them is considered by the Awiakay to be equal to a duel (Hoenigman [22]; see also 64: 12). Among the Awiakay, there are rare instances of women who, after being widowed, took on the men’s role of a hunter and went to the bush to hunt for pigs. One woman, recently deceased, was highly praised for having killed a pig by herself – an achievement that normally marks a boy’s transition to being considered a young man, ready to marry and provide for his family. People continue to recount this case, and her story remains a point of reference in the village. There is no recollection of a woman ever killing a cassowary. But in general, women do not hunt with weapons. They often kill bandicoots though, simply by kicking them with their leg or hitting them with a stick. Their principal domain in terms of animal protein is fishing, as mentioned in the Introduction. Both men and women harvest sago grubs and collect brush turkey eggs. Cassowary eggs are found by chance rather than through deliberate search, but because they are highly valued, encountering one requires an immediate ritual: the finder jumps away, shouting *“Apuria, apuria!”* ‘Wasps, wasps!’ in a great leap, just as they would from a dangerous wasp nest. Only upon returning can the remaining eggs of the clutch become visible. While hunting and fishing magic were abandoned along with male and female initiations in the 1960s, rituals like this one were practised much longer, but are now disappearing with the last generation who held on to them.

In the current study, boys and girls showed a similar increase in Awiakay naming ability with age, but more data are needed for this age group before any clear inferences can be drawn. There were only two girls among the top ten highest scoring children, and this may reflect different gender roles that manifest themselves from a young age. This may also explain the steeper increase in performance among younger and intermediate age groups of the male respondents. Girls and women – young and old – stay in the vicinity of the village or bush camps, whereas boys and men spend more time in the forest on hunting expeditions. As mentioned in the previous section, boys tend to become independent earlier, whereas girls stay closer to home (and to their mothers), helping with the care of younger siblings and domestic tasks.

### The gold rush

As indicated in the Introduction, the Awiakay language enjoyed high vitality prior to the gold rush, used in all kinds of social contexts, and by all Awiakay. Tok Pisin was present both as a lingua franca, used with occasional visitors from other villages, and as a language of authority, mainly in church, but also in public speeches and quarrels. It was also used for all the novelties coming to the village from the outside world, although the Awiakay quickly coined their own terms, to use in their ‘hidden talk’, a lexical substitution register used to hide their true intentions from outsiders [28]. While both adults and children were bilingual in Awiakay and TP, children started speaking Awiakay and acquired TP later (Table 1). It was not until 2024, six years after the discovery of gold and four years into the gold rush, that the first author first noticed some children conversing entirely in TP, and not being able to understand Awiakay.

As mentioned in the Introduction, the data presented here only give indirect indications of a potential adverse effect of the gold rush on TEK transmission in Kanjimei. Gold has drawn in everyone from Kanjimei, but a few families have managed to limit their involvement by spending longer periods in the village, rather than at the gold camps. There appears to be a negative correlation between the intensity of engagement with gold extraction and the ability of younger Awiakay to name plants and animals in their native language. Both boys and girls with greater direct or indirect exposure to gold extraction performed poorly, and were more likely to name plants and animals in TP. Simultaneously, there were occasional mitigating factors that have a protective effect on TEK transmission. These include delayed or reduced exposure to gold extraction, the need to be self-sufficient (as in the case of F4), or the presence of caregivers who are motivated to teach TEK and associated language. There is also some impressionistic evidence that children of consultants who worked with the first author over the course of her fieldwork (such as M4) tended to perform better. It is likely that the first author’s persistent questions, and the consultants’ having to reflect on various interconnected topics (including traditional beliefs and practices, TEK and language) and explain these complex issues to the former, may have caused these families to develop an increased awareness of the value of ancestral knowledge, and rekindled a curiosity about such matters among some younger Awiakay.

The strategies used by children with limited knowledge of the Awiakay ethnobiological lexicon are worth pointing out. Some started generalising the best-known animal or plant from a group of related ethnotaxa, and using it for all members of that group. In many cases, this is a novel phenomenon, that did not exist in the ethnoclassification systems of older speakers. In the case of bats, for example, many younger respondents used the name *anyŋgwaŋ* ‘Bismarck flying fox’ (*Pteropus neohibernicus*) as if it were a generic term for all bats, even though Awiakay has at least three other names for different bat species. This strategy is not surprising, given the local linguistic context. It mirrors an Awiakay organising principle: the name of one creek or mountain range becomes a ‘head-name’, and all other place names stem from it [22]. In the same way, *ayŋgwaŋ* has become a ‘head term’ for bats.

At the moment, there seems to be a tendency for children with significant exposure to gold mining to know fewer Awiakay names of plants and animals. Future studies, carried out with a larger number of younger participants, should be able to quantitatively verify if this is a real pattern. A longitudinal study would also be of immense value in determining the learning trajectories of the children already included in the present study. Such a study would show if the low-scoring children have indeed been adversely affected long-term by the gold rush, or if they are able to acquire the relevant linguistic and ethnobiological knowledge at a later point in their lives. It would also be interesting to see if they are able to fill the lexical gaps cause by the attrition of Awiakay with new or existing TP vocabulary, but this possibility seems unlikely (cf 65).

### Changing interactions with plants and animals

The gender- and age-related differences evident in Tables 3 and 4 can be attributed to a range of factors, including some that were in effect well before the gold rush. The large and prominent Blyth’s Hornbill’s behaviour played a prominent role in initiation ceremonies, but these have not been performed since the 1960s. Hornbills can still be seen flying overhead, but at a great height. Greatly reduced interaction with this bird may explain why around a third of the boys and girls could not name it. In the case of smaller birds, such as the Zoe Imperial Pigeon or bats, such as the Dark Sheath Tailed Bat, men and boys could identify and name these ethnospecies more readily probably because they are killed and eaten opportunistically while hunting for larger game, such as wild pigs. Hunting of wild animals other than pigs was already somewhat marginal in the lead up to the gold rush (as Sillitoe [66] observed among the Wola), but older Awiakay remember a time when birds, bats and marsupials used to make a greater contribution to people’s protein intake in the days before the missionaries (as Dwyer [67] noted for the Etolo). The first author was shown specialised spears for hunting birds and possums during her first visit to Kanjimei in 2004; these were last used in the 1990s, and no one engages in this form of hunting nowadays.

Some ethnospecies are currently known to a lesser extent by women and children because their natural habitat lies far from the village. Marsupials such as Brown’s Pademelon or the liana *wapukam* (with edible fruit) are found in the mountains or foothills respectively. These ethnospecies would only be encountered by men on hunting trips, or by women and children during time spent at bush camps closer to the mountains. Children, in particular, are now unfamiliar with these ethnotaxa, due to time spent with their caregivers at gold camps.

While gardens have largely been abandoned, some people still plant *makayp* (*Derris* vine for fish poisoning) despite the gold rush. They are even selling it to one another, as they need fish, both for themselves, but also for selling in gold camps where food is always in high demand. Any kind of food, along with tobacco, betel nut, pepper betel and lime can easily be sold to gold prospectors.

As mentioned earlier, the most important animal hunted is wild pig. There are several different ways of hunting pigs, the most frequent being *tay yawa* ‘sago trap’ whereby a pig is lured to an opened sago trunk where it is speared at night by a hunter in a hide built of sago leaves. If the wounded pig escapes, it is followed by dogs and killed when it weakens. Since the beginning of the gold rush in the early 2020s, a practice called ‘hiring a dog’ was instituted, whereby a person who is in need of a pig will rent a dog (along with its owner) to go hunting at night. One might also ‘hire a gun’ (along with its owner) for the same purpose. Prior to the gold rush, men would go hunting themselves, but in times of gold, they no longer have the time or inclination for that activity. Many prefer to keep searching for gold, hoping to find enough to be able to afford to buy meat or even hire a dog or a gun. The same holds true of sago. While a day’s worth of pounding sago can sustain a family for a few days, a day’s yield of gold (if one is lucky), might buy more sago, as well as some other desirable goods – unless the gold is stolen, demanded for a compensation, or spent otherwise before it can be used for food.

### Limitations and future directions

This study has brought to light some intriguing results, but a few limitations should also be kept in mind. First, the lack of a ‘control’ village against which to compare Kanjimei might seem problematic, but this was unavoidable, as the entire Awiakay population lives within a single village. Moreover, this was a population-level study, focusing on demographic factors, rather than on ecological or geographic variables, which might necessitate comparisons between different villages. Nevertheless, we acknowledge that a systematic comparison with a similarly remote village (of another ethnic group) that was unaffected by gold mining would have been ideal. We invite other ethnobiologists working in PNG to carry out comparable studies at other locations.

A further limitation is the potential confounding of the effects of the gold rush on the youngest children interviewed and the normal TEK acquisition curve. We have tried to overcome this problem by presenting qualitative life history data for key children, but a follow-up study in a few years, with the same individuals, should provide more definitive results.

The first author’s ongoing *Awiakay-Tok Pisin-English photographic dictionary of Awiakay flora and fauna* may provide a useful resource, should members of the Awiakay community wish, at some point in the future, to strengthen the transmission of TEK. The present study suggests that knowledge relating to birds, bats and other mammals may be particularly vulnerable to attrition, and could therefore benefit from greater attention in the dictionary and other documentation initiatives. Although this finding derives from the Awiakay case, similar patterns may occur in other communities in PNG and perhaps elsewhere. Researchers engaged in the documentation of language and/or TEK in this part of the world may therefore wish to consider giving these taxa the same attention as is normally given to plants.

## Conclusions

The gold rush is not the first time the Awiakay have been caught up in a lucrative resource-gathering frenzy. As mentioned in the Introduction, eaglewood, which Awiakay call *is-kamia* ‘tree-meat’, was collected and sold for more than two decades. Before the year 2000, the Awiakay put no value on eaglewood. When the first author arrived in Kanjimei in June 2004, however, the village was almost empty, as nearly everyone was in the bush harvesting the now-precious resin. Even back then, Awiakay used the money they earned with eaglewood to buy rice, tinned fish, two-minute noodles, soap, lamps and kerosene, petrol, mosquito nets, cooking pots, spoons, knives, bush knives, tools, hooks, fishing nets, clothes, and also outboard motors and radios. However, a trip to harvest eaglewood meant several weeks spent in remote bush camps and on the mountain. Women and children would normally stay in the camps and process sago, while the men would try to kill a pig so that they could then leave some smoked meat with women and children, and carry the rest with them to the mountain. They would stay there as long as their sago lasted. In the mountain they would sleep in rock shelters or build temporary shelters for themselves. But although their primary goal was searching for eaglewood, this did not prevent them from hunting and foraging.

Gold panning is a different type of activity altogether, and has caused many Awiakay families to cease interacting with the natural environment as their ancestors once did. This has led to an alarming decrease in linguistic skills and ethnobiological knowledge among younger children. The analysis presented in this paper suggests that the gold rush is directly responsible for these changes, but further research is required to confirm this claim. It also remains to be seen if the children who performed poorly in our interviews will gradually attain adult-like levels of TEK as they mature, or if their TEK has been permanently impacted. Any lasting breakdown in TEK transmission caused by the gold rush should become apparent (i.e. quantifiable) by the end of the current decade, and we plan further field studies to investigate this possibility.

## Data Availability

The datasets used and/or analysed during the current study are available from the corresponding author on reasonable request.

## Footnotes

1 We label Kanjimei as ‘remote’ based on its distance from the provincial government centres and services, and a complete lack of road access.

2 Larger game animals were hunted with the help of hunting dogs and weapons such as bows and arrows or, nowadays, rifles. Birds and bats were targeted with slingshots, which were introduced during the colonial period.

3 There is both linguistic and cultural evidence [27: 102–104] which supports Paul Roscoe’s [32], hypothesis that the Arafundi people traditionally foraged in the tropical forest, and have only recently begun to supplement their diet of sago, game, insects and plant matter found in the forest with limited gardening.

4 The Awiakay have names for 33 types of bananas, all of which grow on their land, although some have been imported from other areas.

5 Mining has brought significant social and environmental transformations to many rural communities in Papua New Guinea, affecting local livelihoods, land use practices, social relations and human environment interactions [33, 34, 35]. Most existing research has examined these processes in the context of large-scale extractive projects. In contrast, this study focuses on the consequences of artisanal small-scale mining, an increasingly important livelihood activity in many parts of Papua New Guinea [36, 37].

6 The total Awiakay population of 475 (235 female, 240 male) individuals at the time of data collection includes a large number of young children (108 between the ages of 0 and 8 years). There were 16 individuals above the age of 60.

7 This case brings to mind Ian Saem Majnep, a Kalam naturalist who wrote about birds, animals and plants of his Kalam country, Upper Kaironk Valley in the Schrader Range, PNG Highlands, in collaboration with anthropologist and ethnobiologist Ralph Bulmer. Saem gained detailed environmental knowledge as a young boy while living with his widowed mother and foster-sister at the edge of the forest, where he had to provide for them [47: 9, 184].
